# Steroid-Resistant Graves’ Orbitopathy Treated with Tocilizumab in Real-World Clinical Practice: A 9-Year Single-Center Experience

**DOI:** 10.3390/jcm10040706

**Published:** 2021-02-11

**Authors:** José V. Pérez-Moreiras, María Varela-Agra, M. Consuelo Prada-Sánchez, Guillermo Prada-Ramallal

**Affiliations:** 1 Moreiras Ophthalmology Center, International Institute of Orbit and Oculoplastics, 15702 Santiago de Compostela, Spain; moreiras@clinicamoreiras.com (J.V.P.-M.); cprada@clinicamoreiras.com (M.C.P.-S.); 2 Epidemiology, Statistics and Research Methodology Unit, Institute for Health Research Foundation (FIDIS), 15706 Santiago de Compostela, Spain

**Keywords:** tocilizumab, steroid-resistant, Graves’ orbitopathy, Clinical Activity Score, thyrotropin receptor antibody, proptosis, eyelid retraction, diplopia, orbit

## Abstract

This study aimed to assess the effectiveness and safety of tocilizumab use for the treatment of active steroid-resistant Graves’ orbitopathy (GO). A retrospective longitudinal study was conducted by reviewing the medical records at a single center between November 2009 and December 2018. A total of 114 patients with steroid-resistant Graves’ orbitopathy were examined and treated with tocilizumab, of which 54 adults met the inclusion criteria. No concomitant medication for the treatment of orbitopathy was used. The main primary outcomes included changes from baseline in the Clinical Activity Score (CAS) and thyrotropin receptor antibody (TRAb) levels throughout therapy with tocilizumab. The absolute responses to treatment were defined as the achievement of CAS ≤ 1 and TRAb ≤ 10 U/L. A composite ophthalmic score including CAS, proptosis, eyelid retraction, and diplopia was used to evaluate individual improvement in GO. Adverse drug reactions were also assessed. Analysis of the patient’s CAS and TRAb levels showed meaningful reductions during tocilizumab treatment. Differences between values at baseline and subsequent time points were statistically significant (*p* < 0.001 for all comparisons). The absolute CAS response (CAS = 0 or 1) was achieved in 74% (37/50) of patients after the fourth dose of tocilizumab (at week 16), with a TRAb response being achieved in 55% (23/42) of patients. The relative CAS response (reduction ≥ 2 points) was achieved in 90.9% of patients (40/44) after the first dose of tocilizumab (at week 4). Measurements of proptosis (reduction ≥ 2 mm in 78% of patients, 42/54) and eyelid retraction (reduction ≥ 2 mm in 75%, 33/44), and the prevalence of diplopia (improvement in 68%, 19/28) were significantly reduced after the last dose of tocilizumab (*p* < 0.001 for all comparisons). GO improved in 98% (53/54) of patients when at least two criteria of the composite evaluation were required. Four patients exhibited disease recurrence, defined as an increase in CAS of ≥2 points in the six months following the date of inactivation. Most adverse drug reactions were mild or moderate in severity. In conclusion, our data suggest that a course of at least 4 months (one monthly dose) of tocilizumab therapy provides a significant benefit to patients with active moderate-to-severe steroid-resistant GO.

## 1. Introduction

Graves’ orbitopathy (GO) is the main extra-thyroidal manifestation of Graves’ disease, with a prevalence in Europe of about 10 per 10,000 inhabitants [1]. However, the physiopathology of GO remains unclear. In recent years, there has been increasing evidence that thyrotropin receptor and insulin-like growth factor 1 receptor are expressed in the orbital fibroblasts of GO patients and act by stimulating adipogenesis and hyaluronic acid synthesis [2]. Several studies suggest that the thyrotropin receptor antibody (TRAb) plays a central role in the pathogenesis of GO [3,4]. In the active phase of GO, there is a predominant production of pro-inflammatory cytokines such as interleukin-6 (IL-6). The concentration of IL-6 is increased in Graves’ disease patients with active GO compared with those patients with inactive or absent GO [5]. IL-6 might play a role in the pathogenesis of GO by stimulating thyrotropin receptor expression within the orbit [6], and therefore it has been considered a good target for treatment.

A wide range of immunosuppressive therapies have been used to try to reduce the autoimmune and inflammatory phase and stop the natural course of GO [7]. Although corticosteroids are the most widely used treatment in GO, around 20–30% of patients are poorly responsive [8], leading to relapse or progression of GO. For moderate-to-severe GO that does not respond to intravenous pulses of methylprednisolone, the European Group on Graves’ Orbitopathy (EUGOGO) guidelines recommend combination treatment with rituximab [9]; however, randomized clinical trials have reported conflicting results in this regard [10,11].

Patients not responding to intravenous treatment with corticosteroids who are resistant to other therapies (e.g., rituximab) are prone to requiring repeated surgery for exophthalmos, eyelid retraction, and strabismus correction [12,13]. Many of these patients suffer from frequent relapses within a few months after surgery. Among the new therapies against GO, there is teprotumumab, a monoclonal antibody that binds to insulin-like growth factor 1 receptor and induces its degradation [14]. Two clinical trials with teprotumumab have been conducted [15,16], demonstrating the safety and superiority of the drug over a placebo for the treatment of GO. In early 2020, teprotumumab was approved by the United States Food and Drug Administration (FDA) for the treatment of GO.

An alternative therapy is tocilizumab, an anti-IL-6 receptor monoclonal antibody. IL-6 plays an important role in the activation of B cells and the development of antibody-producing plasma cells [17]. Therefore, tocilizumab can reduce the concentrations of memory B cells and immunoglobulin levels [17]. Tocilizumab was first introduced as a treatment for severe glucocorticoid-resistant GO in 2009, with the first results published in a small case series [18]. The efficacy of tocilizumab was later supported by a randomized clinical trial in patients with active steroid-resistant GO [19], as well as by several case reports and case series [20,21,22,23,24,25,26,27,28,29].

In this study, we investigated the effectiveness and safety of tocilizumab under real-world conditions to treat steroid-resistant patients with moderate-to-severe GO.

## 2. Materials and Methods

### 2.1. Study Design and Patients

A retrospective, longitudinal study was conducted on a cohort of patients treated with tocilizumab at Moreiras Ophthalmology Center (Spain), in which each patient acted as their own control. Between November 2009 and December 2018, demographic, clinical, and laboratory data were reviewed from medical records.

Patients were eligible for inclusion in the study if they were over 18 years of age; had moderate-to-severe GO (according to EUGOGO severity classification); had not responded to previous treatment with intravenous corticosteroid infusions; had a Clinical Activity Score (CAS) of 4 or greater on a 10-point scale in the more severely affected eye; and had a time between the diagnosis of orbitopathy and the start of treatment of less than 24 months. Exclusion criteria were the following: treatment with radioactive iodine during the previous 6 months; blood disorders (anemia, neutropenia, or thrombocytopenia); neoplastic disease; cardiovascular or pulmonary disease; uncontrolled diabetes mellitus; positive history of hepatitis B or C liver disease; and pregnancy or lactation.

Systemic and ophthalmic evaluations were done before, during, and after the treatment and follow-up periods. All patients were examined by the same skilled orbital ophthalmologist (J.V.P.-M.). Prior to initiating treatment with tocilizumab, all patients underwent a complete blood test, which included an evaluation of thyroid function and autoimmunity (triiodothyronine, thyroxine, thyroid-stimulating hormone, and TRAb), lipid status and liver function, and a thorough serology examination to rule out the presence of active infectious diseases. All patients had normal thyroid hormone levels at the time of inclusion, and all patients were monitored by their referring endocrinologist during the treatment and follow-up periods. Blood measurements were repeated every 4 weeks.

At least 3 months elapsed from the last steroid treatment in all patients before starting the administration of tocilizumab. This enabled the effects of the first medication on study outcomes to be minimized. Tocilizumab (RoACTEMRA; Roche Pharmaceuticals, Basel, Switzerland) was administered IV at a dose of 8 mg/kg every 4 weeks on an outpatient basis. No medication for the treatment of orbitopathy was given alongside tocilizumab. All patients were administered tocilizumab for least 4 cycles, except for two patients who needed only 3 cycles.

### 2.2. Outcomes and Assessments

The primary study outcomes included changes in the CAS and TRAb levels from baseline throughout the period of treatment with tocilizumab. The overall response to treatment was described based on the following (absolute) criteria for improvement: Patients who had a response were defined as those whose CAS decreased below 2 points (i.e., disease inactivation when CAS = 0 or 1; although a CAS threshold of ≤2 was also considered for a sensitivity analysis), while a decrease below the threshold of 10 U/L (levels within normal range) was considered to define the response with regard to TRAb. The period of disease stabilization was defined as the six months following the date of inactivation (i.e., approximately one month after the date of the last dose of tocilizumab). Recurrence was defined as an increase of CAS of ≥2 points at any time from the date of disease stabilization until the end of the individual follow-up period.

Other primary outcomes included the occurrence of proptosis, eyelid retraction, and diplopia. Proptosis was measured in millimeters using the same Hertel instrument. Upper and lower eyelid retraction was measured in millimeters with respect to the corneal limbus. Subjective diplopia was evaluated in the primary and extreme gaze positions.

A composite ophthalmic score was used to evaluate individual improvement in GO after the last dose of tocilizumab treatment. GO was considered to have improved when at least two of the following (relative) criteria for improvement were met: (A) a reduction in CAS by at least 2 points; (B) a reduction in proptosis of at least 2 mm in at least one eye, with no increase of 2 mm or greater in the contralateral eye; (C) a reduction in upper eyelid retraction by at least 2 mm or greater in at least one eye, with no increase of 2 mm or greater in the contralateral eye; and (D) an improvement in diplopia (i.e., disappearance of diplopia in the primary gaze position or in the extreme gaze position). As a sensitivity analysis, the requirement to meet at least three criteria for GO improvement was additionally considered.

Secondary outcomes were changes in eyelid edema, extraocular motility, visual acuity, and the visual field after treatment with tocilizumab. The intensity of eyelid edema was graded on an increasing visual scale from 0 to 3. Extraocular motility was evaluated as an improvement of 5 or more degrees (9 prism diopters) according to the Hirschberg test. Visual acuity was evaluated using the Snellen test, with 10/10 being considered normal vision. The visual field was measured using a Humphrey field meter.

Safety was also assessed, including adverse drug reactions of special interest.

### 2.3. Statistical Analyses

A sample size of 50 patients was estimated to provide the study with a power greater than 90% to detect a 1-unit within-subject pre–post difference in the CAS (given a standard deviation [SD] of 2) and a power greater than 80% to detect a 25-unit pre–post difference in the TRAb levels (given a SD of 60) or a 30 percentage-point difference in the proportion of patients with diplopia (in extreme gaze). A sample size of 100 eyes was estimated to provide the study with a power greater than 95% to detect a 1-unit within-subject pre–post difference in the occurrence of exophthalmos (given a SD of 3) or upper eyelid retraction (given a SD of 2). However, when analyzing by subgroups, the power could have been lower.

Results of quantitative variables are presented as mean or median (standard deviation or range). When the assumption of normality in the model’s hypotheses failed, non-parametric tests were used. The Wilcoxon matched-pairs signed-rank test was used for within-subject comparison (CAS, TRAb, exophthalmos, eyelid retraction, and visual acuity outcomes). The Mann–Whitney U test was used to compare independent groups (smokers vs. non-smokers). Correlations were assessed using the Spearman’s rank-order coefficient (CAS scores and TRAb levels). Results of qualitative variables are presented as frequencies and percentages. Associations between categorical variables were analyzed using the McNemar or McNemar–Bowker (Chi-square) test to compare related groups (categorized exophthalmos, diplopia, edema, and visual field outcomes).

A post hoc analysis was conducted to better elucidate the TRAb outcome (dependent variable) in subgroups by smoking status. For this purpose, a repeated-measurements mixed model was developed, incorporating the following as explanatory (independent) covariates: a baseline score, smoking status, time (as a repeated measure), and the smoking status by time interaction. The baseline score represented the probability of being a smoker given a patient’s set of baseline covariates (i.e., a propensity score that, due to the limited number of patients exposed, only included the two baseline covariates most strongly associated with the outcome [30]).

We additionally constructed logistic regression models to explore the association between baseline characteristics and improvement in GO after the last dose of tocilizumab treatment, based on the composite ophthalmic score when at least 3 criteria were required for improvement. Odds ratios were calculated to estimate association strengths between each variable at baseline and the outcome.

All statistical tests were two-sided and performed at the level of significance of α = 0.05. The analysis was performed using SPSS 25.0 (IBM Corp., Armonk, NY, USA).

## 3. Results

### 3.1. Patients

A total of 114 steroid-resistant patients with active GO were screened, and 54 patients met the inclusion criteria (mean age 53.8 years, SD = 10.5, 41 of whom were women, 75.9%), with a median follow-up (time from first treatment until last visit) period of 22.0 months (range 10.1–98.1). The median number of doses taken was 4.5 (range 3–9). More than 4 cycles of tocilizumab were necessary for 27 patients (50.0%). A complete description of patients’ baseline characteristics is shown in Table 1.

### 3.2. Primary Outcomes

#### 3.2.1. Clinical Activity Score and Thyrotropin Receptor Antibody

**Figure 1:**
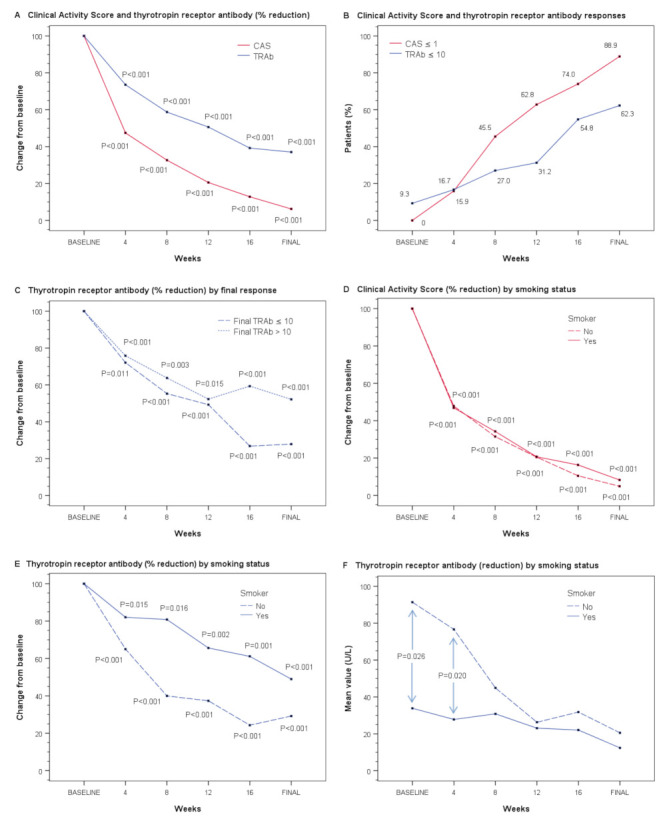
Main effectiveness outcomes during the course of the treatment with tocilizumab. (**A**) Panel A shows the rate of reduction in the Clinical Activity Score (CAS) and thyrotropin receptor antibody (TRAb) level as the mean percentage changes from baseline (mean relative percentage values are shown to facilitate interpretation, while *p*-values were calculated with the Wilcoxon test using the medians of the absolute values of the measurements). CAS and TRAb values correspond to measurements at baseline and at one month after each treatment dose. (**B**) Panel B shows the responses with regard to CAS (defined as CAS ≤ 1) and TRAb (defined as TRAb ≤ 10 U/L) throughout the treatment period (CAS and TRAb responses by smoking status can be seen in Figure S1A,B). (**C**) Panel C shows the mean percentage change in TRAb from baseline according to the final TRAb response. (**D**,**E**) Panels D and E show the rate of reduction in CAS and TRAb, respectively, according to smoking status. (**F**) Panel F shows the mean TRAb levels in smokers and non-smokers; comparisons between groups were performed with the Mann–Whitney U test. (The mean CAS reduction by smoking status can be seen in Figure S1C). The *p*-values shown in panels **C**, **D**, and **E** were calculated with the Wilcoxon test using the medians of the absolute values of the measurements.

The evaluation of the patient’s CASs showed a meaningful reduction after treatment with tocilizumab, from an initial mean CAS of 6.7 points (SD = 1.5) to a final mean CAS of 0.4 points (SD = 0.7). TRAb levels were also reduced after treatment, from initial mean TRAb of 69.0 U/L (SD = 87.5) to final mean TRAb of 17.3 U/L (SD = 40.4). Comparisons during the treatment period indicated that each pairwise difference between the data at baseline and subsequent time points was statistically significant (*p* < 0.001) for both variables. Figure 1A shows the rates of CAS and TRAb reduction over the course of the treatment with tocilizumab as the mean percentage changes from baseline. As can be seen in the figure referenced above, there was a strong positive correlation between the mean absolute CAS scores and TRAb levels over time (Spearman’s rho = 0.9, *p* = 0.005).

The proportions of patients who had a response for each outcome are shown in Figure 1B. The absolute CAS response (CAS ≤ 1) was observed early, with 15.9% (7/44) of patients achieving the response after the first dose (one month from baseline), and a response occurred in an increasing percentage of patients throughout the study (74%, 37/50, after the fourth dose and 88.9%, 48/54, after the last dose, range 3–9). Table S2a details these results over time, and shows whether the criterion considered was CAS ≤ 2. Although 9.3% (5/54) of patients already had normal TRAb levels (≤10 U/L) at baseline, the TRAb response was slow. After the fourth dose, a response was achieved in 54.8% (23/42) of patients (19 of them with CAS ≤ 1), and this increased slightly after the last dose to 62.3% (33/53) of patients (30 of them with CAS ≤ 1) (Table S2b).

When analyzing the TRAb level by subgroups according to the final TRAb response, the results suggest that the rate of reduction diverged from the third dose onwards (Figure 1C). When evaluating the rates of CAS and TRAb reduction in relation to smoking status, the rate of CAS reduction did not differ between smokers and non-smokers (Figure 1D), while the rate of TRAb reduction was slower in smokers (Figure 1E). Although the graphical representation of TRAb percentage reduction visually suggests that there was a difference between smokers and non-smokers, only the TRAb levels of smokers at baseline and at 4 weeks were statistically lower (33.9 U/L, SD = 34.2 vs. 93.6 U/L, SD = 103.9, *p* = 0.026; and 27.8 U/L, SD = 39.2 vs. 76.7 U/L, SD = 94.6, *p* = 0.020, respectively). In fact, the mean TRAb level of smokers always remained below that of non-smokers (Figure 1F and Table S3).

In view of the results obtained above (Figure 1F) and as an additional exploratory analysis, we studied the effect (adjusted and unadjusted by a baseline score) of tobacco consumption, time, and the interaction between tobacco consumption and time on TRAb levels. As expected, since the TRAb levels reduced progressively, the time factor was statistically significant (*p* = 0.003). In addition, tobacco consumption was also significantly related (*p* = 0.002) at the expense of non-smokers (*p* < 0.001) vs. smokers (*p* = 0.826). When the model was adjusted by a baseline score, similar results were obtained.

Table 2 shows the mean baseline and final TRAb levels according to the thyroid status and prior (>6 months) radioactive iodine and thyroidectomy therapy (Figure S1D–F shows the change in the mean TRAb levels over time in relation to these variables). Table S4 shows the mean baseline and final CAS scores.

#### 3.2.2. Recurrences

Four patients (7.4%) exhibited disease recurrence at months 1, 15, 21, and 30, respectively, from stabilization, which required 1, 3, 8, and 2 additional doses of tocilizumab, respectively, to restore normality.

At baseline, the mean CAS of the 4 patients who relapsed was 6.8 points (SD = 1.0) and the mean TRAb was 38.9 U/L (SD = 50.6) (1 patient with levels within the normal range), which reduced to 0 and 6.6 (SD = 1.3) (all patients with normal levels) after the first regime of tocilizumab. At the time of relapse, the mean CAS was 3.5 points (SD = 1.3) and the mean TRAb was 22.6 U/L (SD = 23.1) (1 patient with normal levels); these values improved to 0 and 9.0 (SD = 5.7) (3 patients with normal levels) after the second regime of tocilizumab.

#### 3.2.3. Composite Evaluation of Graves’ Orbitopathy

**Figure 2:**
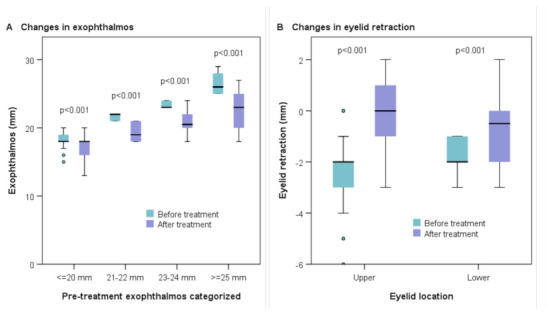
Exophthalmos and eyelid retraction before and after treatment with tocilizumab. The box plots show the changes in exophthalmos (categorized in four groups based on the initial measurement of exophthalmos) (**A**) and eyelid retraction (upper and lower) (**B**) before and after treatment with tocilizumab. *P*-values were calculated with the Wilcoxon test.

The mean initial **proptosis** prior to treatment with tocilizumab was 21.8 mm (range 15–29), which was significantly reduced to 19.5 mm (range 13–27) after the last dose (*p* < 0.001). For better analysis of proptosis, patients were divided into four groups based on the initial exophthalmos measurement (Table 3 and Figure 2A). In Table S5a, it is shown, for example, that 21 eyes had exophthalmos ≥25 mm before treatment, and the number was reduced to 6 after treatment.

Statistically significant reductions in both upper and lower **eyelid retraction** were found after treatment with tocilizumab (*p* < 0.001) (Table 3 and Figure 2B).

Forty-seven patients were assessed for **diplopia** in their primary gaze at baseline, with horizontal, vertical, and mixed strabismus presented in 4 (8.5%), 7 (14.9%), and 1 patient (2.1%), respectively. After treatment with tocilizumab, 3 patients with horizontal strabismus and 4 patients with vertical strabismus achieved complete resolution. In all cases of resolved strabismus, the initial gradation was 5 degrees or lower. Twenty-eight out of 47 patients (59.6%) had diplopia in extreme gaze, and this significantly reduced to 11 patients (23.4%) after treatment (*p* < 0.001).

GO was considered to have improved when at least two of the prespecified criteria (including reduction of CAS, reduction in proptosis, reduction of upper eyelid retraction, and improvement of diplopia) were met. According to this **composite outcome**, GO improved in 98.1% (53/54) of the patients in our series after the last dose of treatment with tocilizumab. However, it should be noted that if the number of criteria required to be met was at least 3, 74.0% (37/50) of patients would have improved. In this case, patients could be classified into two groups according to the number of criteria met: non-responders (1 or 2 criteria, *n* = 13) and responders (3 or 4 criteria, *n* = 37). In Table S6, baseline patient characteristics are compared according to these groups, based on the composite ophthalmic scores under the latter criteria. Factors associated with a good response to the treatment were also explored, with the only significant finding being that prior radioactive iodine therapy multiplied the risk of GO not improving after treatment by 9 (OR = 0.11, 95% CI 0.02–0.53, *p* < 0.01). However, the predictive power of the regression models was quite weak due to the small patient sample size. Therefore, multivariate analysis adjusted by baseline characteristics was not conducted [30].

Regarding CAS, it is noteworthy that 90.9% of patients (40/44) had already experienced a ≥2 point decrease after the first dose of tocilizumab treatment (at week 4) (Table S2c). Figure S2 details the criteria met at the individual level, showing the frequency of each criteria and the frequency of GO improvement after the last dose according to the number of criteria required to be met.

### 3.3. Secondary Outcomes

The occurrence of upper and lower **eyelid edema** also decreased significantly after therapy (*p* < 0.001) (Table S5b). Eleven eyes had maximal edema in the upper eyelid before treatment, and this reduced to zero eyes after treatment. In the case of the lower eyelid, the number of cases went from six to zero.

The occurrence of **extraocular motility** showed significant clinical results. Specifically, upgaze and adduction eye movements presented median improvements of 10 degrees (range 5–20) and 10 degrees (range 5–15), respectively, in 70.4% (38/54) and 75.9% (41/54) of patients.

A loss in **visual acuity** secondary to GO was described in 21.3% (23/108) of the eyes. The mean value prior to treatment was 6.8 out of 10 (range 3.0–9.0), which was significantly improved to 8.1 (range 6.0–10) after treatment (*p* = 0.002). One patient developed dysthyroid optic neuropathy, which resolved without the need for surgery. The **visual field** test was abnormal in 25.5% (24/94) of the eyes evaluated, having normalized in all of them after treatment (*p* < 0.001).

### 3.4. Safety

Adverse drug reactions were observed in 26 patients (48.1%). Hypercholesterolemia was the most frequent adverse effect (14 patients). The development of neutropenia was observed in six patients, leukopenia in four patients and thrombocytopenia in two patients. Hypertransaminasemia was observed in two patients. All other adverse drug reactions occurred in the following order of frequency: asthenia (6), pruritus/urticaria (3), cellulitis (3), and upper respiratory tract infection (1). One case presented with anaphylactic shock with bronchospasm during the perfusion of tocilizumab.

## 4. Discussion

The results suggest that tocilizumab can be used as an effective and safe treatment in patients with active moderate-to-severe steroid-resistant GO disease. All primary and secondary outcomes improved after patients received tocilizumab, with meaningful within-group differences shown in CAS, TRAb, proptosis, eyelid retraction, and diplopia. Thus, most patients improved according to the composite evaluation of GO.

The main finding was a significant reduction in CAS outcomes during treatment with tocilizumab, with inflammation improving from the second month in about half the patients. The reduction in CAS scores was accompanied by a slower rate of decline in TRAb levels, which were normal in almost two-thirds of patients after completing treatment. Levels of autoantibodies remained high in the rest of the patients, despite them being clinically inactive. Dragan et al. [31] argued that the TRAb measurement is an excellent tool for assessing the prognosis and evaluating the response of active GO to treatment [32,33]. The results of the present study corroborate the close correlation between CAS and TRAb [34].

Tobacco has been widely studied as a risk factor for Graves’ disease [35] and active GO [32,36]. **Smoking** increases the risk of developing GO [37], adversely affects prognosis [38,39], and alters the response to treatment [40,41]. In this study sample, the rate of decline in CAS did not vary between smokers and non-smokers. However, smoking status modified the effect on TRAb over time in the initial measurements. Once treatment was initiated, all patients in our series showed reductions in TRAb levels, with the rate of reduction being slower in smokers (Figure 1E), because their baseline levels were already lower (Figure 1F). This might suggest a differential effect of previous corticosteroid therapy on biochemical characteristics between smokers and non-smokers. In fact, smoking is known to reduce the effectiveness of steroid prophylaxis [40]. Under this assumption, among smokers who did not respond to previous steroid use, there would be a subgroup of milder patients (with lower TRAb levels) compared to steroid-resistant non-smokers (with higher TRAb levels), which would explain such behavior. An alternative explanation would be a possible attenuating effect of nicotine through activation of the cholinergic anti-inflammatory system [42,43]. This last hypothesis, however, is counterintuitive and may be conflicting. We also found that prior therapy with radioactive iodine may be a risk factor for GO progression, which is in line with results obtained in other studies [44,45].

GO typically follows a two-phase (active and inactive) course. The duration of the **active phase** varies, according to different authors, from 6 to 36 months [45] and can last for up to 4 years in smokers [46]. For this reason, patients with an active phase of longer than 24 months were excluded from this study. However, in the authors’ experience, many patients with a longer period of active disease also experience improvement with tocilizumab treatment.

Furthermore, previous treatment with **intravenous steroids** did not appear to mask the results obtained, as they had a maximum benefit period of 6 to 12 weeks in patients with moderate-to-severe active GO [47]. All patients included in our study remained untreated for a 3-month period after treatment with steroids without experiencing clinical improvement in their condition.

The clinical and biochemical improvements observed after treatment with tocilizumab were greater than those in the usually described natural history of GO [45,48] and in **placebo groups** of different studies [10,15,16,19] (Table 4 and extended version Table S7). This supports the fact that clinical improvement is attributable to tocilizumab treatment and should not be interpreted as spontaneous improvement.

In terms of low **activity disease**, when considering a threshold of CAS ≤ 2, 86.0% of patients reached the response criterion in our sample after the fourth dose (at week 16). These data are considerably higher than those obtained in the placebo group (35.2%) from the only tocilizumab study with an independent control arm to date (a randomized clinical trial [19]). Even considering a CAS threshold ≤1, the outcome (74.0%) is also clearly superior to those obtained in the placebo groups of both teprotumumab trials [15,16] (19% and 15%, at week 18). However, all of these comparisons and the following should be interpreted with caution given the different settings of each study. Note that, in our series, the response was almost 90% after the last treatment dose (Table S2a). With respect to the **TRAb** outcome, we observed a median decrease of 81% in our sample compared with 33% at week 16 in the placebo group of the rituximab trial (Table 4).

Regarding the individual outcomes included in the **composite score** defined in the present study, all patients showed a decrease in CAS of ≥2 points after the fourth dose (week 16), compared with 58.8% of the patients from the placebo group in the clinical trial with tocilizumab [19]. In the rituximab trial [10], the outcome was even lower (25%) (Table 4). The results obtained in a recently published multicenter study [29] on the use of tocilizumab in steroid-resistant patients are also very favorable.

The absolute reduction in **exophthalmos** was around 10% in our series, which is 3 times greater than the best figures described with any of the placebo groups (3%, in [15]). Regarding the relative response criterion of a reduction of ≥2 mm, 3 out of 4 patients improved after tocilizumab treatment, while the response was always less than 40% with the placebo [10,15,19]. The mean change reduction in **eyelid retraction** was over 90% in each of the eyes in our study, while that obtained in the placebo group of the tocilizumab trial [19] was less than 5%. In terms of relative improvement, 75% of the patients experienced a clinically significant reduction of ≥2 mm after the last dose of tocilizumab. In the placebo groups of the tocilizumab and rituximab trials, the rates of improvement in eyelid retraction were much lower (7% at best [19]), although it should be noted that the criterion considered was a ≥3 mm reduction for the lid aperture or fissure assessment (Table 4).

In both the primary and extreme gaze positions, the prevalence of **diplopia** in our cohort was reduced, while it increased in some placebo groups [16] at the time points closest to 16 weeks from treatment onset. Regarding the response criterion, diplopia improved in 68% of our sample patients, which is a greater percentage than any of the improvements in the placebo groups (Table 4). The criteria used to evaluate diplopia were, however, heterogeneous between studies.

The composite outcome for assessing the overall improvement in GO was also quite different among the studies mentioned. This means that the respective results are not comparable with each other.

To better understand how a disease develops and how it can be addressed, observational studies of the **natural history** of the disease are of interest. These studies follow the course of a disease’s process from its onset to resolution or until the death of the individual (without medical intervention). In fact, a recent FDA draft [49] showed a growing interest in natural history studies of rare diseases, as their knowledge is an important prerequisite that greatly improves the design of clinical trials. However, studies on the knowledge of the natural history of rare diseases are very scarce, the patient samples are small, and mild cases are generally studied (logically untreated). Natural history studies, and especially retrospective studies, are challenging because disease processes are complex and evolve over different chronological periods for different subjects. Furthermore, from methodological and statistical points of view, these studies present numerous limitations and potential biases (e.g., confounding, selection biases such as referral bias and length-biased sampling, measurement biases, immortal time bias, among others [50,51]). Therefore, no conclusion can be drawn about the natural history of moderate-to-severe GO, since it is rapidly treated with disease-modifying therapies. Nevertheless, the results described in the placebo groups of the trials discussed above suggest that the so-called Rundle’s curve may also apply to patients with moderate-to-severe GO [52].

There are probably three therapies with **promising results** for the treatment of moderate-to-severe GO: tocilizumab, teprotumumab, and rituximab. However, patients enrolled in the teprotumumab [15,19] and rituximab [10,11] trials were not steroid-resistant, thus limiting the comparability of these results to those obtained in both the tocilizumab trial [19] and the present observational study. While rituximab has been associated with better results than methylprednisolone [11], the conflicting results reported in the trial with placebo [10] warrant further randomized studies. Rituximab may be a promising drug whose place in the treatment of GO remains unclear for now. Unlike the clinical trial with tocilizumab [19], patients in the present study were able to continue treatment until the disease was considered to have ceased. Therefore, it seems that the results were even more favorable under clinical practice conditions, having obtained a clinically significant mean decrease in exophthalmos of 2.3 mm after the last treatment dose (median number of doses 4.5, range 3–9), higher than the 1.1 mm obtained in the tocilizumab trial (at week 16) and similar to that obtained with teprotumumab [15].

Regarding **recurrences** of GO, there are few published data. Selva et al. [53] collected data on late relapses that appeared after 5 years of stability and found a recurrence rate of 5.0%. Patel et al. [46] assessed the occurrence of relapses after 6 months of stability and found a rate of 15.7%, with most events occurring in the first 5 years. In the present study, in which almost half of the patients were followed for at least 2 years, a minimal stability period of 6 months after treatment was also established to analyze the occurrence of relapse after treatment. Under this criterion, and considering an increase in CAS of at least 2 points as the definition of a recurrence event, the rate was notably lower (7.4%).

Most of the **adverse reactions** were similar to those already known. It should be noted, however, that anaphylactic shock with bronchospasm occurred in one patient, which required the administration of tocilizumab to be stopped.

This study is **limited** by its observational, retrospective, self-controlled nature. In this regard, a single-arm design does not rule out the observed effect being explained by the natural history of the disease or by antithyroid drug therapy, since the conversion from hyperthyroidism to euthyroidism might be associated with the improvement of orbitopathy. Regarding the post hoc exploratory analysis of the TRAb outcome, the use of propensity score methods has been broadly implemented in practice to compare “non-manipulable” conditions or disease states, such as smoking, aiming to adjust for several covariates when the data are retrospectively collected. However, this practice might be conceptually questionable under such (non-manipulable) conditions [54]. The study’s limitations also include the relatively small patient sample size, which might not have been sufficient to draw significant inferences when comparing or modeling by subgroups.

## 5. Conclusions

Despite the limitations of this study, our data suggest that at least four months of treatment with tocilizumab (one monthly dose) provides a significant benefit to patients with active steroid-resistant Graves’ orbitopathy. These data and previous work clearly illustrate the value of including tocilizumab in the management algorithm for moderate-to-severe GO. However, more controlled studies are needed to establish the priority of tocilizumab as a valid therapy in GO treatment guidelines.

## Figures and Tables

**Table 1:** Baseline characteristics of the patients.

Variable	Baseline
Age—*mean yr (SD)*	53.8 (10.5)
Female sex—*% (No. of patients)*	75.9 (41)
Smoker—*% (No. of patients)*	38.9 (21)
Diabetes—*% (No. of patients)* ^1^	9.4 (5)
Thyroid status—*% (No. of patients)* Hyperthyroidism Hypothyroidism	87.0 (47) 13.0 (7)
FT3—*mean pg/mL (SD) (No. of patients)* ^2^	3.4 (1.0) (16)
FT4—*mean ng/dL (SD) (No. of patients)* ^2^	1.3 (0.7) (52)
TSH—*mean mU/L (SD) (No. of patients)* ^2^	3.0 (4.0) (53)
Thyrotropin receptor antibody—*mean U/L (SD) (No. of patients)* Normal TRAb (≤10 U/L)—*% (No. of patients)*	69.0 (87.5) (54) 9.3 (5)
Previous treatment—*% (No. of patients)* Radioactive iodine ^1^ Thyroidectomy	18.9 (10) 24.1 (13)
Time between GO and GD diagnoses—*median mo (range) (No. of patients)* ^3^	4.5 (2.0–11.0) (6)
Time between GD and GO diagnoses—*median mo (range) (No. of patients)* ^3^	5.9 (0.9–200.2) (38)
Time between GO diagnosis and tocilizumab onset—*median mo (range) (No. of patients)*	10.5 (3.5–22.9) (54)
Clinical Activity Score ^4^—*mean pt (SD) (No. of patients)*	6.7 (1.5) (54)
Exophthalmos—*mean mm (SD) (No. of eyes)*	21.8 (3.2) (107)
Eyelid retraction—*mean mm ^5^ (SD) (No. of eyes)* Upper Lower	2.5 (1.3) (88) 1.7 (0.7) (46)
Diplopia—*% (No. of patients) ^6^* Primary gaze Extreme gaze	25.5 (12) 59.6 (28)

Abbreviations: FT3, triiodothyronine (normal range: 2.3–4.2 pg/mL); FT4, thyroxine (normal range: 0.8–1.8 ng/dL); GD, Graves’ disease; GO, Graves’ orbitopathy; mo, months; pt, points; SD, standard deviation; TRAb, thyrotropin receptor antibody (normal range: 0–10 U/L); TSH, thyroid-stimulating hormone (normal range: 0.55–4.78 mU/L); yr, years. ^1^ One patient with a missing value. ^2^ The mean post-treatment values can be found in Table S1. ^3^ Ten patients were excluded due to the coincidence between the dates of diagnosis of GO and GD. ^4^ The Clinical Activity Score is based on a scale of 10 items, each of which is scored as present or absent (1 or 0, respectively): spontaneous orbital pain, pain with extraocular movement, eyelid edema, eyelid erythema, conjunctival hyperemia, chemosis, inflammation of caruncle or plica; and an increase of ≥2 mm in proptosis, decrease in visual acuity of ≥1 Snellen lines, or a decrease in ocular motility in any direction during a period of 1–3 months. ^5^ In absolute values (actual measurements with negative values: ≤0 mm for the upper eyelid and ≤−1 mm for the lower eyelid). ^6^ Forty-seven patients were assessed for diplopia.

**Table 2:** Thyrotropin receptor antibody (TRAb) levels before and after treatment with tocilizumab according to thyroid-related baseline characteristics.

Variable	No. of Patients	Pre-Treatment Mean U/L (SD) ^1,5^	Post-Treatment Mean U/L (SD) ^2,5^	Reduction U/L (%) ^3^	*p*-Value ^4^
Thyroid status ^6^					
Hyperthyroidism	46	58.3 (63.0)	11.4 (10.9)	46.9 (80.4)	<0.001
Hypothyroidism	7	146.4 (172.1)	56.1 (106.1)	90.3 (61.7)	0.018
Radioiodine ^7^					
No	42	71.2 (83.7)	11.7 (11.2)	59.5 (83.6)	<0.001
Yes	10	68.5 (112.9)	41.9 (89.7)	26.6 (38.6)	0.005
Thyroidectomy ^6,8^					
No	40	58.1 (74.0)	17.4 (45.8)	40.7 (70.0)	<0.001
Yes	13	106.3 (117.9)	17.2 (16.2)	89.1 (83.8)	0.002

Abbreviations: SD, standard deviation. ^1^ Baseline measurement. ^2^ Final measurement (one month after the last treatment dose). ^3^ Reductions in absolute value and percentage with respect to the pre-treatment value. ^4^ Mean values are shown to facilitate interpretation, while *p*-values were calculated with the Wilcoxon test (which compares sample medians from both groups). ^5^ The average baseline and final TRAb levels did not differ statistically significantly between the categories of each variable, according to the Mann–Whitney U test (*p* > 0.05 for all comparisons). ^6^ One patient with a missing value for at least one of the variables of interest. ^7^ Two patients with a missing value for at least one of the variables of interest. ^8^ One patient received both therapies (radioiodine and thyroidectomy), in which pre-treatment TRAb = 15.1 U/L and post-treatment TRAb = 7.0 U/L.

**Table 3:** Exophthalmos and eyelid retraction before and after treatment with tocilizumab.

Variable	No. of Eyes	Pre-Treatment Mean mm (range)	Post-Treatment Mean mm (range)	Reduction mm (%) ^1^	*p*-Value ^2^
Exophthalmos					
≤20 mm	40	18.4 (15–20)	17.2 (13–20)	1.2 (6.5)	<0.001
21–22 mm	18	21.6 (21–22)	19.2 (18–21)	2.4 (11.1)	<0.001
23–24 mm	28	23.5 (23–24)	20.8 (18–24)	2.7 (11.5)	<0.001
≥25 mm	21	26.5 (25–29)	22.7 (18–27)	3.8 (14.3)	<0.001
Eyelid retraction					
Upper ^3^	88	−2.5 (−6 to 0)	−0.2 (−3 to +2)	2.3 (92.0)	<0.001
Lower ^4^	46	−1.7 (−3 to −1)	−0.7 (−3 to +2)	1.0 (58.8)	<0.001

^1^ Reduction in absolute value and percentage with respect to the pre-treatment value. ^2^ Mean values are shown to facilitate interpretation, while *p*-values were calculated with the Wilcoxon test (which compares sample medians from both groups). ^3^ Eyes with a pre-treatment value of >0 mm were excluded from the analysis. ^4^ Eyes with a pre-treatment value of >−1 mm were excluded from the analysis.

**Table 4:** Comparison between the results of this study and the placebo groups used in previous clinical trials with monoclonal antibodies in Graves’ orbitopathy.

Characteristic	Stan et al. (2015) [10]	Smith et al. (2017) [16]	Pérez-Moreiras et al. (2018) [19]	Douglas et al. (2020) [15]	Pérez-Moreiras et al. (2021)
Design (study drug)	RCT (rituximab)	RCT (teprotumumab)	RCT (tocilizumab)	RCT (teprotumumab)	Retrospective (tocilizumab)
Control group—No. of patients (type of control)	12 (placebo)	44 (placebo)	17 (placebo)	42 (placebo)	54 (pre–post)
Main outcomes	CAS	Composite outcome (exophthalmos, CAS)	CAS	Exophthalmos, CAS	CAS, TRAb, exophthalmos, eyelid retraction, diplopia
Definition of response	Decrease in CAS of ≥2 pt (24 wk) + decrease in exophthalmos of ≥2 mm, decrease in eyelid fissure width of ≥3 mm, improvement in diplopia score, among others (24, 52 wk) (secondary outcomes) + no need for additional therapy (success)	a) Decrease in exophthalmos of ≥2 mm (24 wk)+ b) decrease in CAS of ≥2 pt CAS ≤ 1 (6, 12, 18, 24 wk)	Decrease in CAS of ≥2 pt (16 wk) *+* decrease in exophthalmos ≥2 mm, decrease in eyelid aperture ≥3 mm, improvement in diplopia score or in signs of soft tissue involvement (16, 40 wk) (secondary composite outcome—at least 2 criteria) CAS ≤ 2 (16, 40 wk)	a) Decrease in exophthalmos of ≥2 mm (24 wk)+ b) decrease in CAS of ≥2 pt (secondary outcome) CAS ≤ 1 (6, 12, 18, 24 wk)	CAS ≤ 1 (also ≤2); TRAb ≤ 10 U/L (4, 8, 12, 16, >16 ^1^ wk) At least 2 (also 3) of the following criteria (≥16 wk): (a) decrease in CAS of ≥2 pt; (b) decrease in exophthalmos of ≥2 mm; (c) decrease in eyelid retraction of ≥2 mm; (d) improvement in diplopia
∆ CAS—mean (SD) (% reduction)	−1.5 (28%) at wk 24 −1.0 (19%) at wk 16 (extrapolated)	−2.4 (46%) (extrapolated) at wk 24 −2.1 (40%) (extrapolated) at wk 18	−3.0 (1.9) (55%) ^2^ at wk 16	n/a	−5.8 (1.5) (87%) at wk 16
Response—No. of patients (%)	Improvement of ≥2 pt in 3/12 (25%) at wk 24	CAS ≤ 1 in 21% at wk 24 CAS ≤ 1 in 15% at wk 18 (extrapolated)	CAS ≤ 2 in 6/17 (35%) at wk 16 Improvement of ≥2 pt in (10/17) 59% at wk 16	CAS ≤ 1 in 21% at wk 24 CAS ≤ 1 in 19% at wk 18	CAS ≤ 1 in 37/50 (74%) at wk 16 CAS ≤ 2 in 43/50 (86%) Improvement of ≥2 pt in 50/50 (100%)
∆ TRAb—median U/L (IQR) (% reduction ^3^)	−9.5 IU/L (49%) at wk 24 (extrapolated) ^4^ −6.5 IU/L (33%) at wk 16 (extrapolated)	n/a	n/a	n/a	−26.0 (−70.4 to 10.4) (81%) at wk 16
Response—No. of patients (%)	n/a	n/a	n/a	n/a	TRAb ≤ 10 in 23/42 (55%) at wk 16
∆ Proptosis—mean mm (SD) (% ^3^)	Left: 0.0 (1.9) (<1%) ^5^ at wk 52 Right: 0.0 (1.8) (<1%) ^5^	−0.5 (2%) ^6^ at wk 24 −0.1 (<1%) ^6^ at wk 18 (extrapolated)	Left: +0.1 (1.7) (<1%) ^2,6^ at wk 16 Right: +0.1 (1.3) (<1%) ^2,6^	−0.5 (2%) ^6^ at wk 24 −0.6 (3%) ^6^ at wk 18	Left: −2.4 (2.1) ^6^ (11%) at wk ≥16 Right: −2.2 (1.8) ^6^ (10%)
Response—No. patients (%)	Improvement of ≥2 mm in 4/12 (33%) at wk 24	n/a	Improvement of ≥2 mm in 2/14 (14%) ^2^ at wk 16	Improvement of ≥2 mm in 4/42 (10%) ITT at wk 24 (4/34, 12%, PP); 14% ITT at wk 18	Improvement of ≥2 mm in 42/54 (78%) at wk ≥16
∆ Eyelid retraction—median mm (IQR) / mean (SD) (% ^3^)	Left: 0.5 (−1.0 to 1.8) at wk 24 ^7,8^ Right: −0.5 (−1.0 to 1.8) ^7,8^	n/a	Left: −0.4 (1.1) (3%) at wk 16 ^2,7^ Right: −0.5 (2.0) (4%) ^2,7^	n/a	Left: 2.0 (1.0-3.0) / 2.3 (1.4) (92%) ^9^ at wk ≥16 Right: 2.0 (1.0–3.0) / 2.4 (1.3) (96%) ^9^
Response—No. of patients (%)	Improvement of ≥3 mm in 0% at wk 24 (extrapolated)	n/a	Improvement of ≥3 in 1/14 (7%) ^2^ at wk 16	n/a	Improvement of ≥2 mm in 33/44 (75%) at wk ≥16
∆ Diplopia—median score (IQR) or % difference ^10^	0.0 (−0.8 to 0.0) ^11^	−2% in extreme gaze at wk 24 4% in primary gaze (increase)	0.0 (−1.0 to 0. 0) ^2,11^	n/a	−36% in extreme gaze at wk ≥16 −15% in primary gaze
Response—No. of patients (%)	Improvement in 8% at wk 24 (extrapolated) ^12^	Improvement in 10/39 (26%) at wk 24 ^13^	Improvement in 0/17 (0%) at wk 16 ^14^	Improvement in 8/28 (29%) at wk 24; 21% at wk 18 ^13^	Improvement in 19/28 (68%) at wk ≥16 ^15^
Overall response (composite outcome)	Success rate of 25% at wk 24	9/45 (20%) ITT; 8/36 (22%) PP at wk 24	5/17 (29%), if ≥2 criteria required (of 5) at wk 16	3/42 (7%) at wk 24 12% at wk 18	53/54 (98%), if ≥2 criteria required (of 4) at wk ≥16 (37/50, 74%, if ≥3 criteria)

Shown are the results obtained in the placebo groups of four clinical trials conducted on monoclonal antibodies for patients with Graves’ orbitopathy at the time point closest to 16 weeks from treatment onset (or the only time point with available data). The present study is also included for comparison. Abbreviations: CAS, Clinical Activity Score; IQR, interquartile range; ITT, intention-to-treat analysis; n/a, characteristic not assessed or measurement not available; PP, per-protocol analysis; pt, points; RCT, randomized controlled trial; TRAb, thyrotropin receptor antibody; wk, weeks. ^1^ Time of the final measurement (after the last treatment dose). ^2^ According to the individual data presented in the supplemental material of [19]. ^3^ Percentage with respect to the pre-treatment value. ^4^ Difference of medians (the median, IQR, of the change was −0.25, −9.8 to 0, according to [10]). ^5^ Proptosis measured with CT scans. ^6^ Proptosis measured with Hertel/Krahn exophthalmeters. ^7^ Lid fissure/aperture width. ^8^ Median (IQR) of the change (% reduction is not interpretable because the baseline data available in [10] are expressed with a different descriptive statistic); the results of our study are expressed in the same way. ^9^ Upper eyelid retraction with a pre-treatment value of ≤0. ^10^ Post–pre percentage difference. ^11^ Diplopia score using the Gorman scale. ^12^ Defined as a decrease in the diplopia score from 3 or 4 to 0, 1 or 2. ^13^ Defined as a decrease of one grade or more. ^14^ Improvement in Bahn/Gorman diplopia score or at least 8 grades. ^15^ Disappearance of diplopia in primary gaze position or in extreme gaze position.

## Data Availability

The data presented in this study are available upon request from the corresponding author.

## Supplementary Materials

The following are available online at https://www.mdpi.com/2077-0383/10/4/706/s1, Figure S1. Main effectiveness outcomes over the course of the treatment with tocilizumab (supplement). Figure S2. Criteria included in the composite ophthalmic outcome at the individual level, according to smoking status and recurrence. Table S1. Thyroid hormone levels before and after treatment with tocilizumab. Table S2a. Absolute response of the Clinical Activity Score (CAS) over the course of the treatment with tocilizumab according to the threshold considered to define disease inactivation. Table S2b. Thyrotropin receptor antibody (TRAb) response over the course of the treatment with tocilizumab. Table S2c. Relative response of the Clinical Activity Score (CAS) over the course of the treatment with tocilizumab. Table S3. Clinical Activity Score (CAS) and thyrotropin receptor antibody (TRAb) levels before and after treatment with tocilizumab according to smoking status. Table S4. Clinical Activity Score (CAS) before and after treatment with tocilizumab according to thyroid-related baseline characteristics. Table S5a. Changes in exophthalmos after treatment with tocilizumab. Table S5b. Changes in edema after treatment with tocilizumab according to eyelid location. Table S6. Comparison of patients’ baseline characteristics according to improvement in Graves’ orbitopathy (response) based on the composite ophthalmic score when at least 3 criteria were required. Table S7. Comparison between the results of this study and the placebo groups from previous clinical trials with monoclonal antibodies in Graves’ orbitopathy (extended version).
