# Supplementary material for: Steroid-Resistant Graves’ Orbitopathy Treated with Tocilizumab in Real-World Clinical Practice: A 9-Year Single-Center Experience
Source: J Clin Med. 2021 Feb 11;10(4):706. doi: 10.3390/jcm10040706 (PMC7916878; doi:10.3390/jcm10040706)
Supplement: Supplementary file 1 [file jcm-10-00706-s001.pdf]

Supplementary file

Steroid-resistant Graves' orbitopathy treated with tocilizumab in real-world clinical practice: a 9-year single-center experience

Journal: Journal of Clinical Medicine

José V. Pérez-Moreiras [1], María Varela-Agra [1,*], M. Consuelo Prada-Sánchez [1] and Guillermo Prada-Ramallal [2,*]

[1] Moreiras Ophthalmology Center. International Institute of Orbit and Oculoplastics. 15702 Santiago de Compostela, Spain

[2] Epidemiology, Statistics and Research Methodology Unit. Institute for Health Research Foundation (FIDIS). 15706 Santiago de Compostela, Spain

* Correspondence: mariavarelaagra@gmail.com (M.V.-A.); prada.ramallal@gmail.com (G.P.-R.)

Supplemental figures

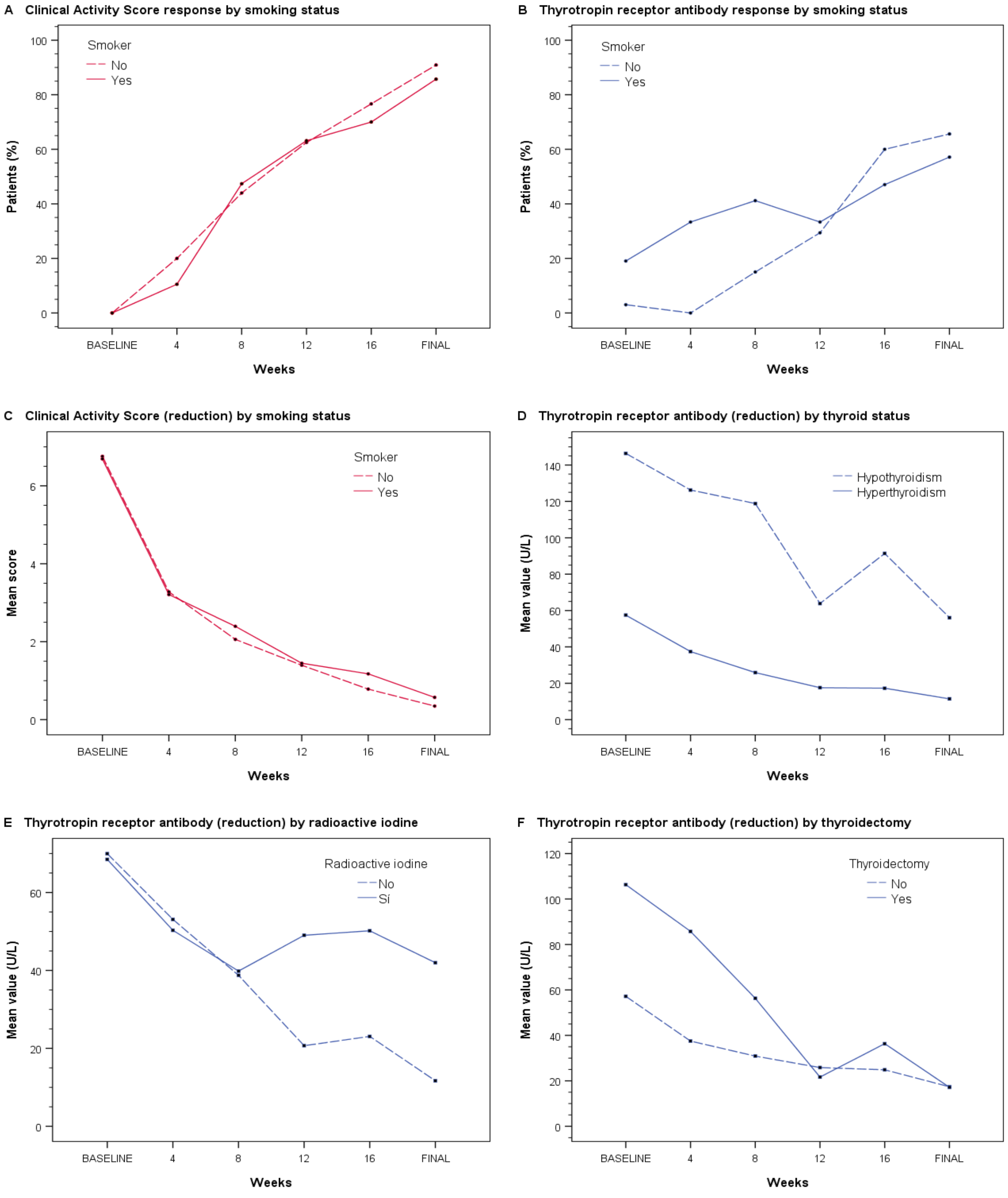

Figure 1. Main effectiveness outcomes during the course of the treatment with tocilizumab (supplement). **(A, B)** Panels A and B show the responses with regard to Clinical Activity Score (CAS) (defined as CAS ≤ 1) and thyrotropin receptor antibody (TRAb) (defined as TRAb ≤ 10 U/L) throughout the treatment period, respectively, according to smoking status. CAS and TRAb values correspond to measurements at baseline and at one month after each treatment dose. **(C)** Panel C shows the mean CAS in smokers and non-smokers. **(D, E, F)** Panels D, E, and F show the mean levels of TRAb according to thyroid status, previous radioactive iodine and thyroidectomy therapy, respectively. Missing data on the outcome variables CAS and TRAb at the intermediate measurements (at 4, 8, and 12 weeks) should be taken into account for the interpretation of these figures. This is especially notable for the TRAb outcome at 12 weeks (Tables S2a and S2b show the number of patients with valid data at each time point).

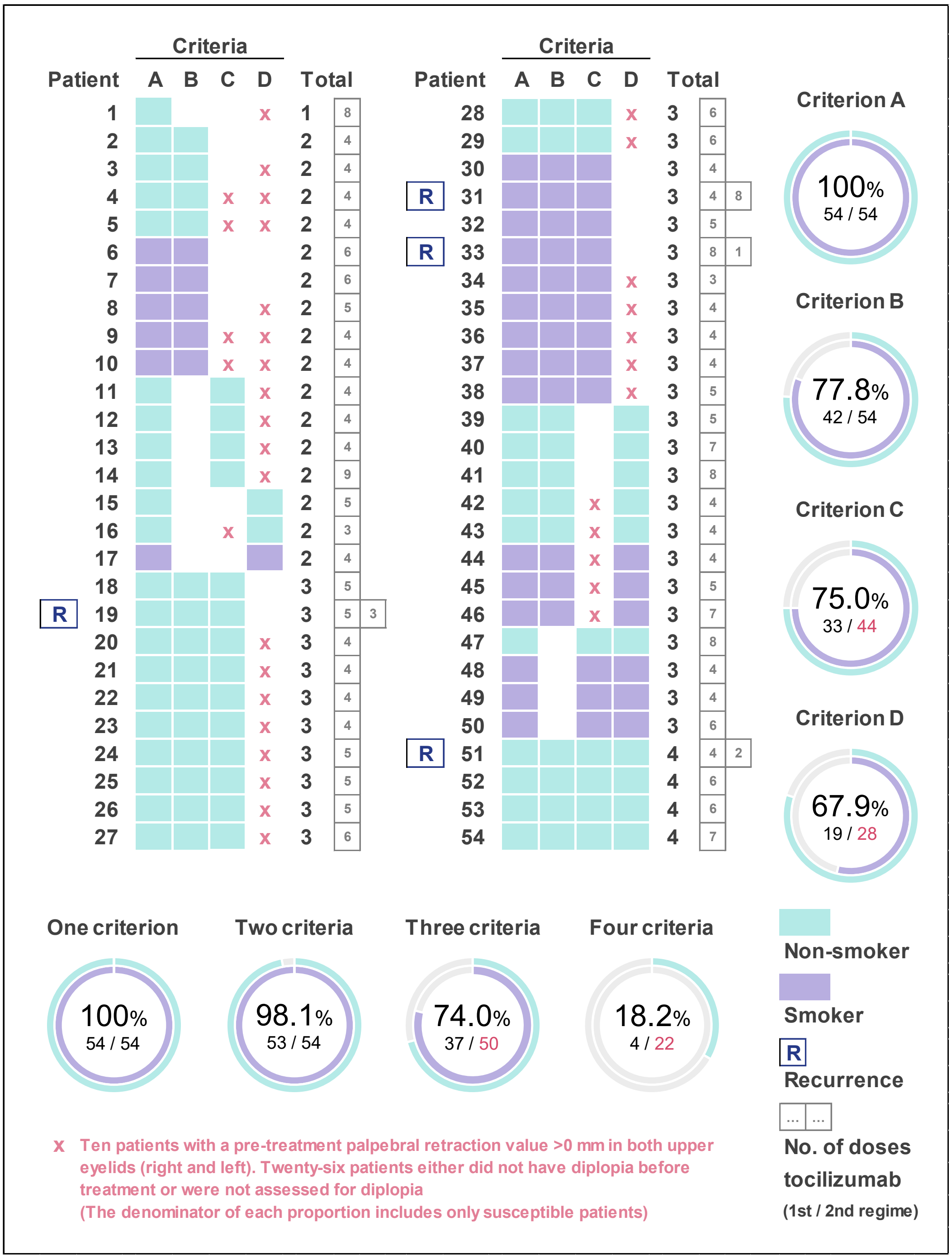

Figure S2. Criteria included in the composite ophthalmic outcome at the individual level, according to the smoking status and recurrence. The charts on the right show the absolute and relative frequency of each criterion used to evaluate the improvement of Graves' orbitopathy (GO). The concentric circles visually represent the percentages corresponding to smokers and non-smokers. The lower graphs show the absolute and relative frequency of GO improvement according to the threshold considered (i.e., the number of required criteria to be met). The following criteria were evaluated at baseline and after the last dose of tocilizumab treatment: **(A)** a reduction of Clinical Activity Score by at least 2 points; **(B)** a reduction in proptosis of at least 2 mm in at least one eye, with no increase of 2 mm or greater in the contralateral eye; **(C)** a reduction of upper eyelid retraction by at least 2 mm or greater in at least one eye, with no increase of 2 mm or greater in the contralateral eye; and **(D)** an improvement in diplopia (i.e., disappearance of diplopia in the primary gaze position or in the extreme gaze position).

Table S1. Thyroid hormone levels before and after treatment with tocilizumab. [1.]

Variable	Pre-Treatment [2]	Post-Treatment [3]	*p*-Value [4]
FT3—*mean pg/mL (SD) (no. of patients)*	3.38 (1.00) (16)	3.17 (0.50) (10)	0.646
FT4—*mean ng/dL (SD) (no. of patients)*	1.26 (0.65) (52)	1.28 (0.38) (52)	0.521
TSH—*mean mU/L (SD) (no. of patients)*	2.96 (4.06) (53)	2.51 (1.76) (53)	0.392

Abbreviations: FT3, triiodothyronine (normal range: 2.3–4.2 pg/mL); FT4, thyroxine (normal range: 0.8–1.8 ng/dL); SD, standard deviation; TSH, thyroid-stimulating hormone (normal range: 0.55–4.78 mU/L). [1] Forty-nine patients (out of 54, 90.7%) were receiving antithyroid treatment at the onset of tocilizumab therapy: 16 (29.6%) patients with hormone therapy, 29 (53.7%) with antithyroid drugs, and 4 (7.4%) with both types of treatment. [2] Baseline measurement. [3] Final measurement (one month after the last treatment dose). [4] Mean values are shown to facilitate interpretation, while *p*-values were calculated with the Wilcoxon test (which compares sample medians from both groups).

Table S2a. Absolute response of the Clinical Activity Score (CAS) during the course of the treatment with tocilizumab according to the threshold considered to define disease inactivation.

Outcome	Baseline	4 Weeks	8 Weeks	12 Weeks	16 Weeks	Final
CAS ≤ 1 points						
% (no. of patients)	0 (0)	15.9 (7)	45.5 (20)	62.8 (27)	74.0 (37)	88.9 (48)
CAS ≤ 2 points						
% (no. of patients)	0 (0)	34.1 (15)	61.4 (27)	81.4 (35)	86.0 (43)	98.1 (53)
Total no. of patients [1]	54	44	44	43	50	54

[1] Number of patients with valid (non-missing) values.

Table S2b. Thyrotropin receptor antibody (TRAb) response during the course of the treatment with tocilizumab.

Outcome	Baseline	4 Weeks	8 Weeks	12 Weeks	16 Weeks	Final
TRAb ≤ 10 U/L						
% (no. of patients)	9.3 (5)	16.7 (6)	27.0 (10)	31.3 (10)	54.8 (23)	62.3 (33)
Total no. of patients [1]	54	36	37	32	42	53

[1] Number of patients with valid (non-missing) values.

Table S2c. Relative response of the Clinical Activity Score (CAS) during the course of the treatment with tocilizumab.

Outcome	Baseline	4 Weeks	8 Weeks	12 Weeks	16 Weeks	Final
Δ CAS ≥ 2 points						
% (no. of patients)	0 (0)	90.9 (40)	93.2 (41)	100 (43)	100 (50)	100 (54)
Total no. of patients [1]	54	44	44	43	50	54

[1] Number of patients with valid (non-missing) values.

Table S3. Clinical Activity Score (CAS) and thyrotropin receptor antibody (TRAb) levels before and after treatment with tocilizumab according to smoking status.

Variable	No. of Patients	Pre-Treatment Mean (SD) [1,5]	Post-Treatment Mean (SD) [2,5]	Reduction *(%)* [3]	*p*-Value [4]
CAS—*points*					
Non-smoker	33	6.8 (1.7)	0.4 (0.6)	6.4 (94.1)	<0.001
Smoker	21	6.7 (1.3)	0.6 (0.9)	6.1 (91.0)	<0.001
TRAb—*U/L* [6]					
Non-smoker	32	93.6 (103.9)	20.6 (51.5)	73.0 (78.0)	<0.001
Smoker	21	33.9 (34.2)	12.4 (10.2)	21.5 (63.4)	<0.001

Abbreviations: SD, standard deviation. [1] Baseline measurement. [2] Final measurement (one month after the last treatment dose). [3] Reduction in absolute value and percentage with respect to the pre-treatment value. [4] Mean values are shown to facilitate interpretation, while *p*-values were calculated with the Wilcoxon test (which compares sample medians from both groups). [5] Baseline TRAb levels of smokers were statistically smaller (p = 0.026). The average baseline CASs, final CASs, and final TRAb levels did not differ statistically significantly between the categories, according to the Mann–Whitney U test ($p > 0.05$ for all comparisons). [6] One patient with a missing value for at least one of the variables of interest.

Table S4. Clinical Activity Score (CAS) before and after treatment with tocilizumab according to thyroid-related baseline characteristics.

Variable	No. of Patients	Pre-Treatment Mean pt (SD) [1,5]	Post-Treatment Mean pt (SD) [2,5]	Reduction pt (%) [3]	*p*-Value [4]
Thyroid status					
Hyperthyroidism	47	6.7 (1.6)	0.4 (0.7)	5.9 (93.7)	<0.001
Hypothyroidism	7	6.7 (1.1)	0.7 (1.1)	5.0 (89.3)	0.017
Radioiodine [6]					
No	43	6.8 (1.5)	0.3 (0.6)	6.5 (95.6)	<0.001
Yes	10	6.4 (1.4)	0.9 (1.4)	5.5 (85.9)	0.004
Thyroidectomy [7]					
No	41	6.5 (1.4)	0.5 (0.8)	6.0 (92.3)	<0.001
Yes	13	7.5 (1.6)	0.2 (0.4)	7.3 (97.3)	0.001

Abbreviations: pt, points; SD, standard deviation. [1] Baseline measurement. [2] Final measurement (one month after the last treatment dose). [3] Reduction in absolute value and percentage with respect to the pre-treatment value. [4] Mean values are shown to facilitate interpretation, while *p*-values were calculated with the Wilcoxon test (which compares sample medians from both groups). [5] The average baseline and final CAS levels did not differ statistically significantly between the categories, according to the Mann–Whitney U test ($p > 0.05$ for all comparisons). [6] One patient with a missing value for at least one of the variables of interest. [7] One patient received both therapies (radioiodine and thyroidectomy), in which pre-treatment CAS = 5 points and post-treatment CAS = 0 points.

Table S5. a. Changes in exophthalmos after treatment with tocilizumab.

		Post-Treatment Exophthalmos—*No. of Eyes*				
		**≤ 20 mm**	**21-22 mm**	**23-24 mm**	**≥ 25 mm**	**Total**
**Pre-Treatment Exophthalmos —*No. of Eyes***	**≤ 20 mm**	***40***	*0*	*0*	*0*	40
	**21-22 mm**	*13*	***5***	*0*	*0*	18
	**23-24 mm**	*14*	*9*	***5***	*0*	28
	**≥ 25 mm**	*8*	*2*	*5*	***6***	21
	**Total**	75	16	10	6	107 [1]

Contingency table is presented where cells show the number of eyes in each category of exophthalmos, before and after treatment. Exophthalmos reduction was statistically significant (McNemar–Bowker test $p < 0.001$). [1] One eye with a missing value for at least one of the variables of interest.

Table S5. b. Changes in edema after treatment with tocilizumab according to eyelid location.

		Post-Treatment Edema—*No. of Eyes*									
		Upper Eyelid					Lower Eyelid				
		**-**	**+**	**++**	**+++**	**Total**	**-**	**+**	**++**	**+++**	**Total**
**Pre-Treatment Edema —*No. of Eyes***	**-**	***3***	*0*	*0*	*0*	3	***23***	*0*	*0*	*0*	23
	**+**	*51*	***5***	*0*	*0*	56	*41*	***7***	*0*	*0*	48
	**++**	*23*	*9*	***6***	*0*	38	*16*	*11*	***4***	*0*	31
	**+++**	*8*	*3*	*0*	***0***	11	*4*	*2*	*0*	***0***	6
	**Total**	85	17	6	0	108	84	20	4	0	108

Contingency tables are presented where cells show the number of eyes in each category of edema intensity: (-) no edema, (+) minimum edema, (++) medium edema, (+++) maximum edema, before and after treatment, by eyelid location. Edema reduction was statistically significant in both groups (McNemar–Bowker test $p < 0.001$).

Table S6. Comparison of patients' baseline characteristics according to improvement in Graves' orbitopathy (response) based on the composite ophthalmic score when at least 3 criteria were required.

Variable	Non-Responders [1] (COS = 1 or 2), *n* = 13	Responders [2] (COS = 3 or 4), *n* = 37	Univariate Analysis [3,4] OR (95% CI)
Age—*mean yr (SD) (No. of patients)*	54.5 (8.5) (13)	52.9 (10.7) (37)	0.99 (0.93–1.05) $p = 0.63$ ($n = 50$)
Female sex—% *(No. of patients)*	84.6 (11)	75.7 (28)	0.57 (0.11–3.05) $p = 0.49$ ($n = 50$)
Smoker—% *(No. of patients)*	30.8 (4)	40.5 (15)	1.53 (0.39–5.91) $p = 0.53$ ($n = 50$)
Diabetes—% *(No. of patients)* [5]	7.7 (1)	8.3 (3)	1.09 (0.10–11.53) $p = 0.94$ ($n = 49$)
Thyroid status—% *(No. of patients)*			
Hyperthyroidism	69.2 (9)	91.9 (34)	5.04 (0.95–26.69)
Hypothyroidism	30.8 (4)	8.1 (3)	$p = 0.06$ ($n = 50$) [6]
FT3—*mean pg/mL (SD) (No. of patients)*	2.9 (0.3) (4)	3.5 (1.1) (12)	3.21 (0.30–35.03) $p = 0.30$ ($n = 16$)
FT4—*mean ng/dL (SD) (No. of patients)*	1.2 (0.3) (11)	1.3 (0.8) (37)	1.21 (0.36–4.02) $p = 0.76$ ($n = 48$)
TSH—*mean mU/L (SD) (No. of patients)*	5.0 (5.9) (12)	2.4 (3.3) (37)	0.87 (0.75–1.02) $p = 0.07$ ($n = 49$)
Thyrotropin receptor antibodies—*mean U/L (SD) (No. of patients)*	89.9 (112.8) (13)	67.7 (80.8) (37)	1.00 (0.99–1.00) $p = 0.44$ ($n = 50$)
Previous treatment—% *(No. of patients)*			
Radioactive iodine [5]	46.2 (6)	8.3 (3)	0.11 (0.02–0.53) $p < 0.01$ ($n = 49$) *
Thyroidectomy	15.4 (2)	27.0 (10)	2.04 (0.38–10.85) $p = 0.40$ ($n = 50$)
Time between GO and GD diagnoses—*median mo (range) (No. of patients)* [7]	3.0 (0-6.0) (3)	0 (0-11.0) (13)	0.97 (0.70–1.35) $p = 0.87$ ($n = 16$)
Time between GD and GO diagnoses—*median mo (range) (No. of patients)* [7]	5.8 (0-156.0) (11)	3.0 (0-200.2) (33)	1.20 (0.99–1.45) $p = 0.06$ ($n = 44$)
Time between GO diagnosis and tocilizumab onset—*median mo (range) (No. of patients)*	8.0 (4.7-12.3) (13)	11.0 (3.5-17.4) (37)	1.15 (0.97–1.37) $p = 0.10$ ($n = 50$)
Clinical Activity Score—*mean pt (SD) (No. of patients)*	7.1 (1.6) (13)	6.7 (1.5) (37)	0.81 (0.53–1.24) $p = 0.33$ ($n = 50$)
Exophthalmos—*mean mm (SD) (No. of eyes)*	21.2 (3.1) (13)	22.0 (3.2) (37)	1.09 (0.88–1.35) $p = 0.42$ ($n = 50$) [8]
Eyelid retraction—*mean mm (SD) (No. of eyes)* [9] Upper	2.2 (1.0) (12)	2.6 (1.2) (32)	0.70 (0.37–1.32) $p = 0.27$ ($n = 44$) [10]
Lower	1.8 (0.4) (6)	1.5 (0.8) (14)	1.77 (0.43–7.32) $p = 0.43$ ($n = 20$) [10]
Diplopia—% *(No. of patients)* [11] Primary gaze	33.3 (3)	25.7 (9)	0.69 (0.14–3.36) $p = 0.65$ ($n = 44$)
Extreme gaze	66.7 (6)	62.9 (22)	0.85 (0.18–3.97) $p = 0.83$ ($n = 44$)

Abbreviations: CI, confidence interval; COS, composite ophthalmic score; FT3, triiodothyronine; FT4, thyroxine; GD, Graves' disease; GO, Graves' orbitopathy; mo, months; OR, odds ratio; pt, points; SD, standard deviation; TSH, thyroid-stimulating hormone; yr, years. Significant risk factors, * $p < 0.05$. [1] Patients who met 1 or 2 criteria of the composite outcome. [2] Patients who met 3 or 4 criteria of the composite outcome (i.e., at least 3 criteria). [3] The comparison analysis was performed with the 50 patients able to have met at least 3 criteria. [4] OR, likelihood ratio test *p*-value. [5] One patient with a missing value. [6] The reference category was "hypothyroidism". [7] The variable included 10 patients in whom the dates of

diagnosis of GO and GD coincided. [8] The variable "exophthalmos" was incorporated into the model as the mean value of exophthalmos between both right and left eyes. [9] In absolute value (actual measurements with negative values: retraction ≤ 0 mm for upper eyelids and ≤−1 mm for lower eyelids). [10] The variable "eyelid retraction" for each location (upper or lower) was incorporated into the model as the mean value between eyelid retraction of both right and left eyes (when retraction ≤ 0 mm for both upper eyelids or ≤−1 mm for both lower eyelids). [11] Forty-four patients (out of 50) were assessed diplopia.

Table S7. Comparison between the results of this study and the placebo groups from previous clinical trials with monoclonal antibodies in Graves' orbitopathy (extended version).

Characteristic	Stan et al. (2015) [1]	Smith et al. (2017) [2]	Pérez-Moreiras et al. (2018) [3]	Douglas et al. (2020) [4]	Pérez-Moreiras et al. (2021)
Design (study drug)	RCT (rituximab)	RCT (teprotumumab)	RCT (tocilizumab)	RCT (teprotumumab)	Retrospective (tocilizumab)
Control group—no. of patients (type of control)	12 (placebo)	44 (placebo)	17 (placebo)	42 (placebo)	54 (pre–post)
Primary outcomes	CAS	Composite outcome (exophthalmos, CAS)	CAS	Exophthalmos	CAS, TRAb, exophthalmos, eyelid retraction, diplopia
Definition of response	Decrease in CAS of ≥2 pt (24 wk)	• Decrease in exophthalmos of ≥2 mm (24 wk) + • decrease in CAS of ≥ 2 pt<br>CAS ≤ 1 (6, 12, 18, 24 wk)	Decrease in CAS of ≥2 pt (16 wk)<br>CAS ≤ 2 (16, 40 wk)	Decrease in exophthalmos of ≥2 mm (24 wk)<br>CAS ≤ 1 (6, 12, 18, 24 wk)	CAS ≤ 1 (also ≤2); TRAb ≤ 10 U/L (4, 8, 12, 16, >16 [1] wk)<br>At least 2 (also 3) of the following criteria (≥16 wk):<br>• decrease in CAS of ≥2 pt<br>• decrease in exophthalmos of ≥2 mm<br>• decrease in eyelid retraction of ≥2 mm<br>• improvement in diplopia
Secondary outcomes	Composite outcome (24, 52 wk):<br>• decrease in CAS of ≥2 pt<br>• no need for additional therapy (success)<br>Also:<br>• Decrease in exophthalmos of ≥2 mm<br>• decrease in eyelid fissure width of ≥3 mm<br>• improvement in diplopia score<br>• improvement in NOSPECS classification by 1 or 2 classes<br>• lagophthalmos, orbital fat/muscle volume, QoL, adverse events	Exophthalmos, CAS, diplopia, GO-QoL, adverse events	Composite outcome (16, 40 wk) (at least 2 criteria):<br>• decrease in CAS of ≥2 pt<br>• decrease in exophthalmos of ≥2 mm<br>• decrease in eyelid aperture of ≥3 mm<br>• improvement in Bahn/Gorman diplopia score or at least 8 grades<br>• improvement in signs of soft tissue involvement<br>QoL (SF-36, GO-QoL), adverse events	• Decrease in exophthalmos of ≥2 mm (24 wk) + • decrease in CAS of ≥2 pt<br>Exophthalmos, diplopia, GO-QoL, adverse events	Eyelid edema, extraocular motility, visual acuity, visual field, adverse drug reactions
Age—mean yr (SD) or median (IQR)	61.8 (11.0)	54.2 (13.0)	47.5 (41.1–57.4)	51.6 (12.6)	53.8 (10.5)
Female gender—No. (%)	8 (66.7)	36 (82.0)	13 (76.5)	31 (73.8)	41 (75.9)
Smoker—No. (%)	2 (16.7)	18 (40.9)	excluded	8 (19.0)	21 (38.9)
**Before placebo/treatment**					

CAS—mean (SD)	5.3 (1.0)	5.2 (0.7)	5.5 (1.5) [2]	5.3 (1.0)	6.7 (1.5)
TRAb—median U/L (IQR) or % (SD)	19.5 IU/L (2.2–28.8)	435.1 (105.2)	7.5 IU/L (0.5–2.8)	n/a	31.8 (17.3–78.0)
Proptosis—mean mm (SD)	n/a	23.1 (2.9) [3]	21.9 (3.3) [2,3]	23.2 (3.2) [3]	21.8 (3.2) [3]
Proptosis left<br>Proptosis right	23.0 (2.4) [4] / 17.3 (2.6) [4]<br>23.3 (3.8) [4] / 17.2 (3.3) [4]	n/a	22.1 (3.5) [2,3]<br>21.7 (3.2) [2,3]	n/a	22.1 (3.3) [3]<br>21.6 (3.1) [3]
Eyelid retraction—mean mm (SD)	n/a	n/a	12.7 (2.3) [2,5]	n/a	-2.5 (1.3) [6]
Retraction left<br>Retraction right	9.8 (2.0) [5]<br>9.0 (2.7) [5]		12.8 (2.2) [2,5]<br>12.6 (2.5) [2,5]		-2.5 (1.3) [6]<br>-2.5 (1.2) [6]
Diplopia—No. patients (%) or median score (IQR)	2.0 (1.0–3.8) [7]	n/a	2.0 (2.0–3.0) [2,7]	n/a	n/a
Extreme gaze<br>Primary gaze	n/a	8 (18.0)<br>4 (9.0)	n/a	n/a	28 (59.6)<br>12 (25.5)
**After placebo/ treatment**					
CAS—mean (SD)	3.8 (1.4) at wk 24<br>4.3 at wk 16 (extrapolated)	n/a	2.5 (2.2) at wk 16 [2]	n/a	0.9 (1.2) at wk 16
Δ CAS—… (% reduction)	−1.5 (28.3%) at wk 24<br>−1.0 (18.9%) at wk 16 (extrapolated)	−2.4 (46.2%) (extrapolated) at wk 24<br>−2.1 (40.4%) (extrapolated) at wk 18	−3.0 (1.9) (54.5%) [2]	n/a	−5.8 (1.5) (86.6%)
Response—No. patients (%)	Improvement of ≥2 pt in 3/12 (25%) at wk 24	CAS ≤ 1 in 21% at wk 24<br>CAS ≤ 1 in 15% at wk 18 (extrapolated)	CAS ≤ 2 in 6/17 (35.2%) at wk 16<br>Improvement of ≥2 pt in (10/17) 58.8% at wk 16	CAS ≤ 1 in 21% at wk 24<br>CAS ≤ 1 in 19% at wk 18	CAS ≤ 1 in 37/50 (74%) at wk 16<br>CAS ≤ 2 in 43/50 (86%)<br>Improvement of ≥2 pt in 50/50 (100%)
TRAb—median U/L (IQR)	10.0 IU/L at wk 24 (extrapolated)<br>13.0 IU/L at wk 16 (extrapolated)	n/a	n/a	n/a	7.6 (5.0–18.8) at wk 16
Δ TRAb—… (% reduction [8])	−9.5 (48.7%) at wk 24 (extrapolated) [9]<br>−6.5 (33.3%) at wk 16 (extrapolated)	n/a	n/a	n/a	−26.0 (−70.4 to 10.4) (80.8%) at wk 16
Response—No. patients (%)	n/a	n/a	n/a	n/a	TRAb ≤ 10 in 23/42 (54.8%) at wk 16
Proptosis—mean mm (SD)	n/a	n/a	22.1 (3.4) [2,3] at wk 16	n/a	19.5 (2.9) [3] at wk ≥16
Proptosis left<br>Proptosis right	na	n/a	22.4 (3 .5) [2,3]<br>22.0 (3.5) [2,3]	n/a	19.7 (2.8) [3]<br>19.4 (3.0) [3]
Δ Proptosis —… (% reduction [8])	Left: 0.0 (1.9) (<1%) [4] at wk 52<br>Right: 0.0 (1.8) (<1%) [4]	−0.5 (2.2%) [3] at wk 24<br>−0.1 (<1%) [3] at wk 18 (extrapolated)	Left: +0.1 (1.7) (<1%) [2,3] at wk 16<br>Right: +0.1 (1.3) (<1%) [2,3]	−0.5 (2.2%) [3] at wk 24<br>−0.6 (2.6%) [3] at wk 18	Left: −2.4 (2.1) (10.9%) [3]<br>Right: −2.2 (1.8) (10.2%) [3]
Response—No. patients (%)	Improvement of ≥2 mm in 4/12 (33.3%) at wk 24	n/a	Improvement of ≥2 mm in 2/14 (14.3%) [2] at wk 16	Improvement of ≥2 mm in 4/42 (10%) ITT at wk 24 (4/34, 12%, PP); 14% ITT at wk 18	Improvement of ≥2 mm in 42/54 (77.8%) at wk ≥16
Eyelid retraction—mean mm (SD)/median (IQR)	n/a	n/a	12.3 (1.9) [2,5] at wk 16	n/a	−0.2 (1.3) [6] at wk ≥16
Retraction left<br>Retraction right	n/a		12.4 (2.1) [2,5]<br>12.1 (1.7) [2,5]		Left: −0.2 (1.3) [6]<br>Right: −0.1 (1.3) [6]
Δ Eyelid—… (% reduction [8])	Left: 0.5 (−1.0 to 1.8) at wk 24 [5,10]	n/a	Left: −0.4 (1.1) (3.2%) / 0.0 (−1.0 to 0.0) [2,5]	n/a	Left: 2.3 (1.4) (92%) / 2.0 (1.0–3.0) [6]

	Right: –0.5 (–1.0 to 1.8) [5,10]		Right: –0.5 (2.0) (4.1%) / 0.0 (–1.0 to 0.0) [2,5]		Right: 2.4 (1.3) (96%) / 2.0 (1.0–3.0) [6]
Response—No. patients (%)	Improvement of ≥3 mm in 0% at wk 24 (extrapolated)	n/a	Improvement of ≥3 in 1/14 (7.1%) [2] at wk 16	n/a	Improvement of ≥2 mm in 33/44 (75%) at wk ≥16
Diplopia—No. patients (%) or median score (IQR)	2.5 (0–4.0) [7] at wk 24	n/a	1.0 (1.0–2.0) [2,7] at wk 16	n/a	n/a
Extreme gaze<br>Primary gaze	n/a	7 (16.0) at wk 24<br>6 (13.0)	n/a	n/a	11 (23.4) at wk ≥16<br>5 (10.6)
Δ Diplopia—median score (IQR) or % difference [11]	0.0 (–0.8 to 0.0) [7]	–2% in extreme gaze<br>4% in primary gaze (increase)	0.0 (–1.0 to 0. 0) [2,7]	n/a	–36.2% in extreme gaze<br>–14.9% in primary gaze
Response—No. patients (%)	Improvement in 8% at wk 24 (extrapolated) [12]	Improvement in 10/39 (26%) at wk 24 [13]	Improvement in 0/17 (0%) at wk 16 [14]	Improvement in 8/28 (29%) at wk 24 [13]<br>21% at wk 18	Improvement in 19/28 (67.9%) at wk ≥16 [15]
Overall response (composite outcome)	Success rate of 25% at wk 24	9/45 (20%) ITT; 8/36 (22%) PP at wk 24	5/17 (29.4%), if ≥2 criteria required (of 5) at wk 16	3/42 (7%) at wk 24<br>12% at wk 18	53/54 (98.1%), if ≥2 criteria required (of 4) at wk ≥16<br>(37/50, 74%, if ≥3 criteria)
Adverse events/effects —No. patients (%), no. events/effects (severe)	3/12 (25%), 4 AE (1 moderate-to-severe event)	32/44 (73%), 1 severe AE	7/17 (41.2%) with >1 AE, 33 AE	29/42 (69%), 1 severe AE	26/54 (48.1), 1 severe adverse drug reaction

Shown are the results obtained in the placebo groups of four clinical trials conducted on monoclonal antibodies for patients with Graves' orbitopathy at the time point closest to 16 weeks from treatment onset (or the only time point with available data). The present study is also included for comparison. Abbreviations: AE, adverse events; CAS, Clinical Activity Score; GO, Graves' orbitopathy; IQR, interquartile range; ITT, intention-to-treat analysis; n/a, characteristic not assessed or measurement not available; PP, per-protocol analysis; pt, points; QoL, quality-of-life; RCT, randomized controlled trial; SD, standard deviation; TRAb, thyrotropin receptor antibody; wk, weeks; yr, years. [1] Time of the final measurement (after the last treatment dose). [2] According to the individual data presented in the supplemental material of [3]. [3] Proptosis measured with Hertel/Krahn exophthalmeters. [4] Proptosis measured with CT scans. [5] Lid fissure/aperture width. [6] Upper eyelid retraction with a pre-treatment value ≤0. [7] Diplopia score using the Gorman scale. [8] Percentage reduction with respect to the pre-treatment value. [9] Difference of medians (the median, IQR, of the change was –0.25, –9.8 to 0, according to [1]). [10] Median (IQR) of the change (% reduction is not interpretable because the baseline data available in [1] are expressed with a different descriptive statistic); the results of our study are also expressed in the same way. [11] Post–pre percentage difference. [12] Defined as a decrease in the diplopia score from 3 or 4 to 0, 1 or 2. [13] Defined as a decrease of one grade or more. [14] Improvement in Bahn/ Gorman diplopia score or at least 8 grades. [15] Disappearance of diplopia in primary gaze position or in extreme gaze position.

References

Stan, M.N.; Garrity, J.A.; Carranza Leon, B.G.; Prabin, T.; Bradley, E.A.; Bahn R.S. Randomized controlled trial of rituximab in patients with Graves' orbitopathy. *J Clin Endocrinol Metab* **2015**,100,432-41. doi: 10.1210/jc.2014-2572.

Smith, T.J.; Kahaly, G.J.; Ezra, D.G.; Fleming, J.C.; Dailey, R.A.; Tang, R.A.; Harris, G.J.; Antonelli, A.; Salvi, M.; Goldberg, R.A.; et al. Teprotumumab for Thyroid-Associated Ophthalmopathy. *N Engl J Med* **2017**,376,1748-1761. doi:10.1056/NEJMoa1614949.

Perez-Moreiras, J.V.; Gomez-Reino, J.J.; Maneiro, J.R.; Perez-Pampin, E.; Romo Lopez, A.; Rodríguez Alvarez, F.M.; Castillo Laguarta, J.M.; Del Estad Cabello, A.; Gessa Sorroche, M.; España Gregori, E.; et al. Efficacy of Tocilizumab in Patients With Moderate-to-Severe Corticosteroid-Resistant Graves Orbitopathy: A Randomized Clinical Trial. *Am J Ophthalmol* **2018**,195,181-190. doi:10.1016/j.ajo.2018.07.038.

Douglas, R.S.; Kahaly, G.J.; Patel, A.; et al. Teprotumumab for the Treatment of Active Thyroid Eye Disease. *N Engl J Med* **2020**,382,341-352. doi:10.1056/NEJMoa1910434.
